# Discriminative context-aware network for camouflaged object detection

**DOI:** 10.3389/frai.2024.1347898

**Published:** 2024-03-27

**Authors:** Chidiebere Somadina Ike, Nazeer Muhammad, Nargis Bibi, Samah Alhazmi, Furey Eoghan

**Affiliations:** ^1^Department of Computing, Atlantic Technological University, Letterkenny, Ireland; ^2^School of Computing, Pak-Austria Fachhochschule Institute of Applied Sciences and Technology, Haripur, Pakistan; ^3^Department of Computer Science, Fatima Jinnah Women University, Rawalpindi, Pakistan; ^4^Computer Science Department, College of Computing and Informatics, Saudi Electronic University, Riyadh, Saudi Arabia

**Keywords:** camouflage object detection, COD, dataset, feature extraction, benchmark, deep learning, convolutional neural network, artificial intelligence

## Abstract

**Introduction:**

Animals use camouflage (background matching, disruptive coloration, etc.) for protection, confusing predators and making detection difficult. Camouflage Object Detection (COD) tackles this challenge by identifying objects seamlessly blended into their surroundings. Existing COD techniques struggle with hidden objects due to noisy inferences inherent in natural environments. To address this, we propose the Discriminative Context-aware Network (DiCANet) for improved COD performance.

**Methods:**

DiCANet addresses camouflage challenges through a two-stage approach. First, an adaptive restoration block intelligently learns feature weights, prioritizing informative channels and pixels. This enhances convolutional neural networks’ ability to represent diverse data and handle complex camouflage. Second, a cascaded detection module with an enlarged receptive field refines the object prediction map, achieving clear boundaries without post-processing.

**Results:**

Without post-processing, DiCANet achieves state-of-the-art performance on challenging COD datasets (CAMO, CHAMELEON, COD10K) by generating accurate saliency maps with rich contextual details and precise boundaries.

**Discussion:**

DiCANet tackles the challenge of identifying camouflaged objects in noisy environments with its two-stage restoration and cascaded detection approach. This innovative architecture surpasses existing methods in COD tasks, as proven by benchmark dataset experiments.

## 1 Introduction

The idea behind Charles Darwin’s theory of evolution and natural selection is the evolution of prey camouflage patterns and the understanding of animal cognition in a more ecological context. The earliest research on camouflage dates to the last century (Cott, 1940). Research by Thayer (1918) and Cott (1940) comprehensively studied the phenomenon of camouflage. Camouflage is an evolutionary concealment technique to mask objects’ location, identity, and movement in their surrounding environment. For living organisms to adapt to their environment, they require the exhibition of adaptive traits or behavioral strategies better suited to the environment. The combination of these physiological characteristics, such as color, pattern, morphology, and behavior (Gleeson et al., 2018; Stevens and Ruxton, 2019), provides them with some survival advantages by disrupting the visual silhouette of animals or potential predators. Inspired by this important natural phenomenon, humans have made attempts to replicate these patterns in many fields.

As a multidisciplinary study of computer science and evolutionary biology, it has a wide range of applications in practical scenarios, including wildlife preservation and animal monitoring; arts (e.g., recreational art) (Chu et al., 2010; Ge et al., 2018); agriculture (e.g., locust detection to prevent invasion); computer vision and other vision-related areas (e.g., search-and-rescue missions in natural disasters; military target detection and surveillance systems; rare species discovery); medical image analysis [e.g., polyp segmentation (Fan et al., 2020b); lung infection segmentation (Fan et al., 2020c; Wu et al., 2021)], to mention a few.

**Figure 1:**
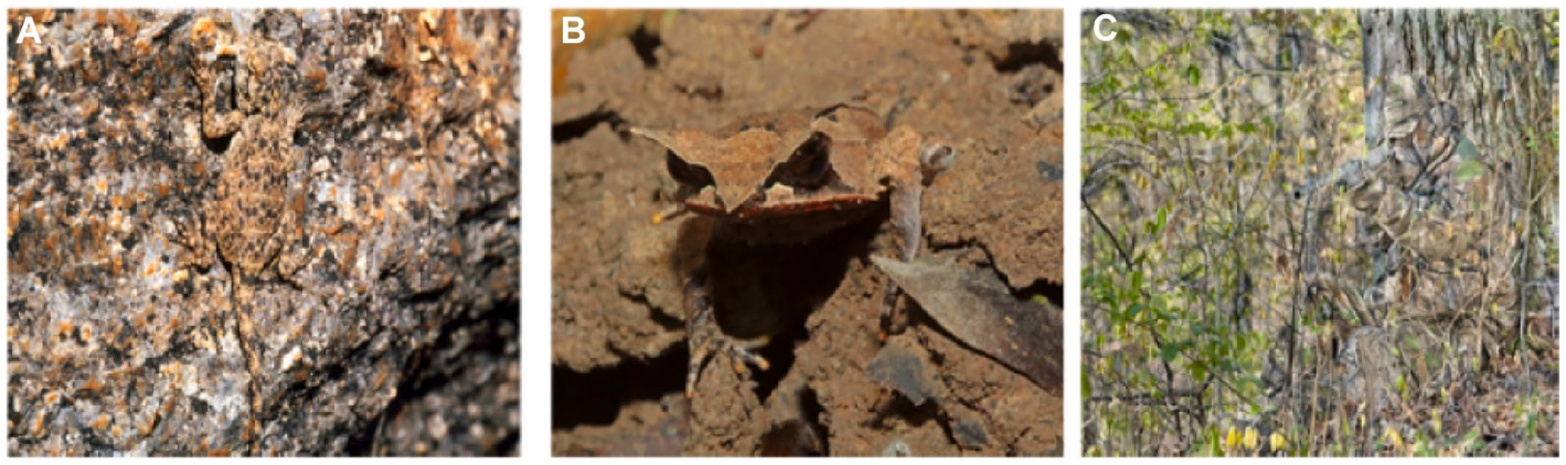
Natural and artificial camouflaged objects. **(A,B)** show Natural camouflage and **(C)** shows Artificial camouflage.

There are two types of camouflaged objects: naturally camouflaged objects and artificially camouflaged objects (Stevens and Merilaita, 2009). Natural camouflage results from the coevolution of predators and prey. Figures 1A,B show disruptive coloration and background pattern matching in animals attempting to exploit predators’ visual processing and cognition. Other camouflage strategies include countershading, transparency, masquerade, distractive markings (Galloway et al., 1802), etc. Artificially camouflaged objects are predatory camouflage strategies often seen in humans, such as military troops, vehicles, weapons, and positions in war zones (Zheng et al., 2018). These objects first observe their environment and elegantly blend their texture patterns to create a familiar scene as the environment to deceive potential observers’ visual perception systems, as shown in Figure 1C.

COD has gained increased attention in the computer vision community but is not well-studied due to the insufficiency of large training datasets and a standard benchmark like Pascal-VOC (Everingham et al., 2015), ImageNet (Deng et al., 2009), MS-COCO (Lin T. Y. et al., 2014), etc.

**Figure 2:**
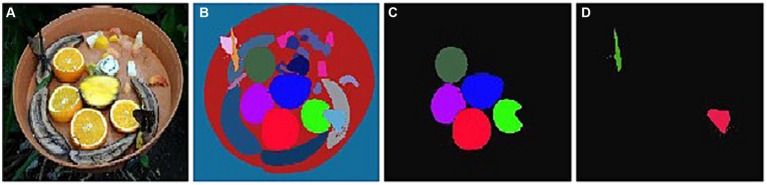
Object segmentation exemplars: **(A)** Given input image, **(B)** GOD, **(C)** SOD, **(D)** COD.

The majority of computer vision literature is largely concerned with the detection/segmentation of non-camouflaged objects (Ren et al., 2017). Based on the detecting and segmenting viewpoint (Zhao Z. Q. et al., 2019), the objects can be divided into three categories: generic objects, salient objects, and camouflage objects. Generic object detection (GOD) is a popular direction in cognitive computer vision which aims to find common objects. They can either be salient or camouflaged. Salient object detection (SOD) aims to find attention-grabbing objects in an image, i.e., objects with pre-defined classes. There exists a vast amount of research works for both generic (Shotton et al., 2006; Liu et al., 2010; Girshick et al., 2014; Everingham et al., 2015; Girshick, 2015; Ren et al., 2015; Kirillov et al., 2019; Le et al., 2020), and salient object detection (Wang et al., 2017; Wu et al., 2019; Zhao J. X. et al., 2019; Zhao and Wu, 2019; Fan et al., 2020a; Qin et al., 2020; Waqas Zamir et al., 2021). COD aims to identify objects whose shape and outline are not easily recognizable in images, as shown in Figure 2. The high intrinsic similarities between the camouflaged objects and the background require a significant amount of visual perception knowledge, hence making COD far more challenging than the conventional salient object detection or generic object detection (Ge et al., 2018; Zhao Z. Q. et al., 2019; Zhao J. X. et al., 2019).

In this paper, we present a review of deep-learning object detection from a camou-flaged perspective. We proposed a discriminative context-aware network called “Di-CANet.” In consideration of the noisy interference in natural systems, the low-frequency distribution contains smooth data disordering while the high-frequency details get an unwanted approximation. These contain channel-wise and pixel-wise features unevenly distributed across the camouflaged image and should be differentiated using weighted information to get an appropriate representation of salient features of objects. Therefore, rather than directly assigning equal weights to the channel-wise and pixel-wise features (Woo et al., 2018), inspired by Qin et al. (2020), we introduced an adaptive restoration block (ARB). This is used to adaptively learn the weights of the image features and assign different weights to them. This not only contributes to the representative ability of convolutional neural networks (CNN) but also provides the required robustness against various types of information preservation. After processing the ARB, these features are complementary-aware according to the fusion pipeline to generate restored camouflage images. Next, a cascaded detection module (Fan et al., 2020a) fortified with a modified receptive field block (Liu and Huang, 2018) was adopted to segment ecological signals and drive the segmentation performance of the target objects during the detection stage. Furthermore, a more refined camouflaged object prediction map is attained with clear boundaries and the generation of an accurate saliency map in terms of contextual details.

With the above considerations, the proposed DiCANet is used to develop a good, camouflaged prediction map. Our contributions can be summarized as follows: (1) We proposed a discriminative context-aware network (“DiCANet”) for camouflage object segmentation; (2) We intelligently infused an adaptive restoration block into a bio-inspired cascaded detection block to effectively guide detection and segmentation performance. The ARB comprises three key components: (a) feature attention block (FAB), (b) Group architecture incorporation, and (c) Attention-based feature fusion network. Details of these components will be discussed in subsequent sections; (3) The proposed COD model boosted performance to a new state-of-the-art (SOTA). The experiments are verified for the effectiveness of our proposed method.

## 2 Related work

This section reviews related works in two folds: image restoration approaches and deep learning-based COD approaches.

### 2.1 Image restoration

Visual information present in the real world contains undesired image contents, and as a positionally sensitive problem, it requires pixel-to-pixel correspondence between the input and the output image. To recover image content from natural images, the traditional approach showed promising reconstruction performance but suffered from computational drawbacks (Ulyanov et al., 2018). Recently, a deep-learning-based restoration model has led to the breakthrough of the conventional approach and achieved state-of-the-art results (Waqas Zamir et al., 2021; Zamir et al., 2022). Designing algorithms robust enough to maintain a spatially precise, high-resolution representation with strong semantic information throughout the entire network has been a challenge. Research by Zamir et al. (2020) proposed a novel multi-scale residual block to effectively learn enriched features for effective real image restoration and enhancement. Despite recent major advancements, state-of-the-art methods suffer from high system complexity, making them computationally inefficient (Nah et al., 2017; Abdelhamed et al., 2018; Chu et al., 2021). To reduce the inter-block complexity of the other SOTA methods (Chen et al., 2022) adopted the stacked neural networks in UNet architecture with skip connections (Ronneberger et al., 2015), following (Wang et al., 2022; Zamir et al., 2022), etc., to design a nonlinear activation-free network framework that is based on CNN rather than a transformer-based network due to SOTA performance drawbacks as reported by Liu et al. (2022) and Han et al. (2021). Research by Qin et al. (2020) proposed a feature fusion attention network, that fuses the FAB with an attention-based multipath local residual structure to focus on learning weights of important spatial information to generate accurate results.

### 2.2 COD

Research into COD has rooted history in biology and arts (Thayer, 1918; Cott, 1940). The studies are still relevant in widening our knowledge of visual perception. The recognition of camouflaged objects has not been well explored in the literature. Early camouflage research focused on detecting the foreground region even when the foreground texture resembled that of the background (Galun et al., 2003; Song and Geng, 2010; Xue et al., 2016). Based on cues such as color, shape, intensity, edge, and orientation, these works distinguished the foreground and background. To address the issue of camouflage detection, a few techniques based on hand-crafted features such as texture (Sengottuvelan et al., 2008; Pan et al., 2011; Liu et al., 2012) and motion (Hou, 2011; Le et al., 2019) are put forth. However, due to the high similarity between the foreground and background, none of these approaches performs well in real application scenarios for segmenting camouflaged objects but is only effective in the case of a simple and non-uniform background. Despite the numerous CNN-based object detection models available, unique designs are required to build models for COD. In contrast to pixel-level segmentation, GOD detects objects with bounding boxes. Furthermore, the segmentation in COD is based on saliency from a human perspective, not semantics, which is not modeled in GOD models. On the other hand, models that are designed for SOD are unable to effectively detect concealed objects. SOD models do non-semantic segmentation and model saliency; nevertheless, they do not specialize in finding indefinite boundaries of objects, as salient objects tend to be of potential human interest. Researchers have proposed several feasible methods for COD.

Recently, (Le et al., 2019) proposed an end-to-end network for segmenting camouflaged objects by integrating classification into the segmentation framework. Research by Lamdouar et al. (2020) and Zhu et al. (2021) has proposed novel approaches based on the assumption that camouflaged objects exist in an image, which is not always practical in the real world. To simulate the real world, (Le et al., 2021) proposed camouflaged instance segmentation without any assumption that camouflaged objects exist in an image. Following the same motivation, (Fan et al., 2020a) proposed a Search Identification Network (SINet) comprising two modules, namely a search module and an identification module, where the former searches whether a potential prey exists while the latter identifies the target animal. The SINet framework leverages a modified Receptive Field Block (Liu and Huang, 2018) to search for camouflaged object regions. Furthermore, aside from their COD model, (Fan et al., 2020a) presented a large COD dataset, called COD10K, which progressed COD research to a new level in the field of computer vision. Similarly, (Dong et al., 2021) proposed an MCIF-Net framework that integrates a large receptive field and an effective feature aggregation strategy into a unified framework to extra rich context features for accurate COD. In addition to existing literature, recent advancements, and relevant studies, such as the notable works of (Hussain et al., 2021; Qadeer et al., 2022; Naqvi et al., 2023), contribute to the understanding of object detection, tracking, and recognition in various contexts, enhancing the breadth and depth of the related literature. Despite research devoted to the challenges in the field of COD to achieve out-standing performance in terms of accuracy, existing deep learning-based COD methods suffer major limitations such as weak boundaries (i.e., edges), low boundary contrast, variations in object appearances, such as object size and shape, leading to unsatisfactory segmentation performance (Fan et al., 2020a; Mei et al., 2021; Ji et al., 2022), and raises the demands of more advanced feature fusion strategies.

Biological studies (Stevens and Merilaita, 2009; Merilaita et al., 2017; Rida et al., 2020) have shown that targets that are deliberately hidden cause more noisy inferences in the visual perception system, which contributes to object concealment. In nature, this is a common phenomenon. Finding ecologically relevant signals hidden in extreme situations becomes a challenge. More so, without precise control of the feature fusion process, detectors are vulnerable to significant attacks from low-frequency details, which cause vague object boundaries and misjudgment in extreme situations. Inspired by this real-world phenomenon, this paper aims to design a novel baseline model to balance the accuracy and efficiency of COD by adaptively exploiting the semantic and spatial information to obtain plausible final context-aware camouflage prediction maps with refined edge boundaries.

## 3 Materials and methods

### 3.1 Motivation and proposed framework

**Figure 3:**
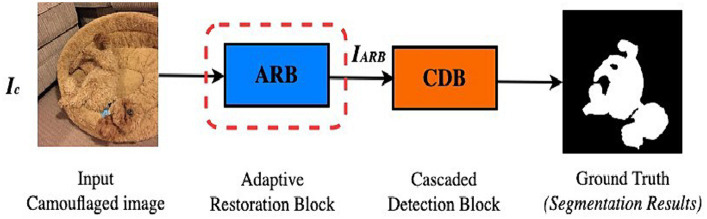
Proposed DiCANet architecture.

The term “survival of the fittest” was conceptualized by Charles Darwin’s theory of evolution (Flannelly, 2017). The survival of numerous species in the wild depends on cultural adaptation; thus, hunting in a wide variety of ecosystems of living things is essential to help organisms thrive in their environment. Motivated by the first two stages of predation, i.e., search (a sensory mechanism) and identification in nature, the DiCANet framework is proposed. The simplified version of the proposed framework is shown in Figure 3. Details of each component are discussed in subsequent sections.

### 3.2 Camouflaged image

The art of camouflage hinges on manipulating an object’s visual appearance to blend into its surroundings. At the heart of this strategy is the concept of pixel similarity. Digital images including those used in camouflage analysis are represented by pixels —tiny blocks of varying features that collectively form the image. In the context of input camouflaged images, the concept of pixel similarity measures how closely the pixels of objects in the camouflaged image match with the surroundings in terms of color, visual patterns, surface variations, and intensity (Talas et al., 2017). The more similar the pixels of the camouflaged object are to those of its intended background (Figure 3), the more effective the camouflage and the harder for observers to spot detectable features of the concealed object. Furthermore, any detectable discrepancies in pixel similarity will reveal the presence of the hidden object, undermining the effectiveness of the camouflage. By analyzing these features and strategically manipulating the pixel attributes of a camouflaged object, we proposed an effective Context-aware Network for Camouflaged Object Detection.

### 3.3 Adaptive restoration block (ARB)

**Figure 4:**
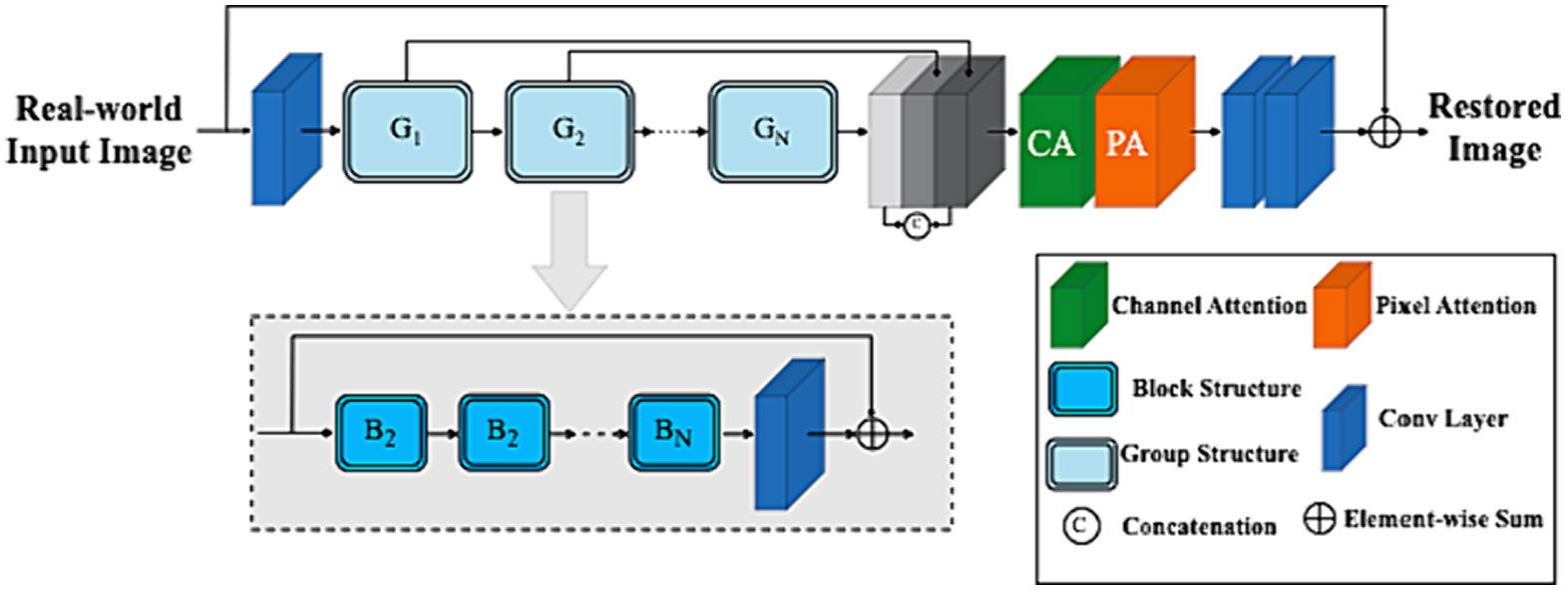
Adaptive restoration block architecture.

To restore concealed images, redundant information unevenly distributed across a real-world image should be adaptively bypassed while robustly allowing the network architecture to focus on more effective information. The ARB framework’s internal block contains several key elements, including (a) the feature attention block (FAB), (b) the attention-based basic block structure, and (c) the feature fusion framework. A detailed framework is shown in Figure 4.

Given a 3−D real-world camouflage input image $I_{c}\in R^{H\;x\;W\;x\;C_{\mathrm{in}}},\mathrm{where}\;H,W$ and $C_{\mathrm{in}}$ are the shape of the image (i.e., dimensions and input channel number) respectively. To map the input camouflaged image space into a higher dimensional feature space, a 3∗3 convolution $H_{SF}\left(\cdot \right)$ was applied to extract shallow features with edge information $F_{sf}\in R^{H\;x\;W\;x\;C}$ formulated as:


(1) $$F_{sf}=H_{SF}\left(I_{c}\right)$$

Deep features $F_{df}\in R^{H\;X\;W\;X\;C}$ are then extracted from $F_{sf}$ as:


(2) $$F_{df}=H_{DF}\left(F_{sf}\right)$$

Where $H_{DF}\left(\cdot \right)$ is the deep features extraction module and it contains K residual Group Architectures block (RGAB) with multiple skip connections. More specifically, intermediate features $F_{1},F_{2},\ldots \ldots ..F_{K}$ and output deep features $F_{DF}$ are extracted block by block as:


(3) $$F_{i}=H_{RGAB_{i}}\;\left(F_{i-1}\right),i=1,2,\ldots \ldots \ldots K,F_{DF}=H_{\mathrm{CONV}}\left(F_{K}\right)\text{,}$$

Where $H_{RGAB_{i}}\left(\cdot \right)$ represents the i−th RGAB and $H_{\mathrm{CONV}}$ is the last convolutional layer, which introduces the convolution operation’s inductive bias into the network and sets the stage for shallow and deep feature aggregation.

### 3.4 Feature attention block (FAB)

**Figure 5:**
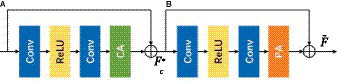
Feature attention block. **(A)** Channel attention (CA). **(B)** Pixel attention (PA).

To improve model representation, an attention mechanism has been introduced inside a CNN (Zhang et al., 2018; Dai et al., 2019; Niu et al., 2020). Many image restoration networks treat channel-and pixel-level features equally, making them incapable of efficiently handling images with uneven low-and high-frequency distributions. Realistically, redundant information is unevenly distributed across images, and the weight of the unwanted pixels should be significantly different for each channel-and pixel-wise feature. In the attention block, features are learned via a dynamic mechanism that enables the model to concentrate on diverse segments of the input data, highlighting pertinent features and attenuating or suppressing irrelevant ones. This process is typically realized through computing attention weights, which signify the significance or relevance of various input features. This adaptive learning approach provides additional flexibility for the network hierarchy in dealing with different types of information. Feature Attention blocks consist of a residual block with channel attention (RB-CA) and residual attention with pixel attention (RB-PA) as shown in Figure 5. The former ensures that different channel features have different weighted information (He et al., 2010) while the latter attentively focuses on informative features in the high-frequency pixel regions.

#### 3.4.1 Channel attention (CA)

To achieve channel-wise weighting for each channel in feature maps, global average pooling (GAP) was employed before feeding the data into fully connected layers for classification tasks. The concept of GAP in CNNs focuses on each feature map (channel) and aggregates information across the entire spatial extent of the feature maps, resulting in a single value per channel (Lin M. et al., 2014; Forrest, 2016; Hu et al., 2018; Machine Learning Mastery, 2019). The 1D vector (channel descriptors) obtained from GAP can then be used in subsequent calculations to extract meaningful features from the image. The mathematical expression detailing how channel descriptors achieve weighted information is as follows:


(4) $$g_{c}=H_{p}\left(F_{c}\right)=\frac{1}{H\;x\;W}\;\sum_{i=1}^{H}\sum_{j=1}^{W}X_{c}\left(i,j\right)$$

Where $H_{p}$ represents the global pooling function, $F_{c}$ the input, and $X_{c}\left(i,j\right)$ denotes the value of c−thchannel $X_{c}$ at spatial position $\left(i,j\right)$. The shape of the feature map changes from CxHxW to Cx1x1 i.e., collapsing HxW. These feature maps are fed through two convolution layers and a computationally efficient sigmoid, followed by ReLu activation function (Figure 5A) to provide the weights of the different channels formulated as follows:


(5) $$CA_{c}=\sigma \left(\mathrm{Conv}\left(\delta \left(\mathrm{Conv}\left(A_{c}\right)\right)\right)\right)$$

Where σ and δ represent the sigmoid function and the ReLu activation function, respectively. By elementwise multiplication of the input $F_{c}$ and weights of the channels $CA_{c}$, the output of the channel attention $F_{c}^{*}$ can be deduced as follows:


(6) $$F_{c}^{*}=CA_{c}⊗F_{c}$$

#### 3.4.2 Pixel attention (PA)

To capture fine-grained details about spatial context, pixel attention (PA) mechanisms actively focus on specific pixels within the entire area (spatial extent) of the feature maps. The concept of attention mechanisms in CNNs, including those that focus on pixel-level details, has been explored in various research studies (e.g., Ismail Fawaz et al., 2019; Dosovitskiy et al., 2020). Inspired by CA (Hu et al., 2018) and spatial attention (SA) (Woo et al., 2018), PA is used to improve the feature representation capacity to obtain images with clear object boundaries. Comparable to CA as shown in Figure 5B, the input $F_{c}^{*}$ (i.e., the output of the channel attention block) is fed through two convolution layers with ReLu and sigmoid activation function (Figure 5B). The shape of the feature map changes from CxHxW to 1xHxW.


(7) $$PA=\sigma \left(\mathrm{Conv}\left(\delta \left(\mathrm{Conv}\left(F_{c}^{*}\right)\right)\right)\right)$$

Recall that activation maps are often followed elementwise through an activation function such as ReLU. Therefore, by elementwise multiplication of $F_{c}^{*}$ and PA, Feature Attention Block (FAB) output $\tilde{F}$is given by:


(8) $$\tilde{F}=F_{c}^{*}⊗PA$$

Integrating Channel Attention and Pixel Attention within CNNs empowers the network to learn both the overall image context and the finer details of specific regions simultaneously. This leads to stronger and more informative feature representations, improving the network’s ability to distinguish objects. Recent research (e.g., Hu et al., 2018; Ismail Fawaz et al., 2019; Dosovitskiy et al., 2020) has explored this combined approach to enhance CNN performance in various computer vision tasks like image classification, object detection, and semantic segmentation.

### 3.5 Block structure (BBS)

**Figure 6:**
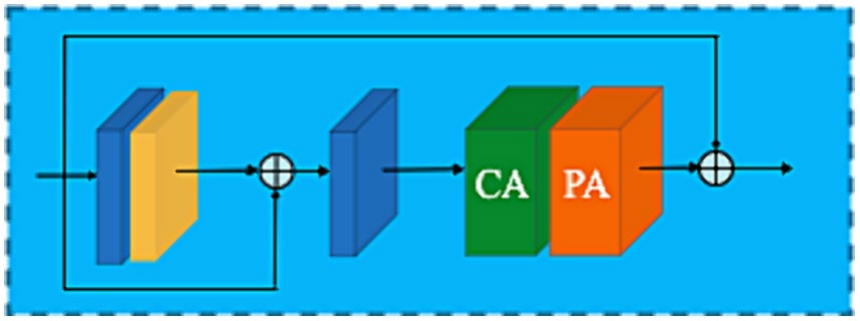
Basic block structure.

The performance of neural networks has been significantly impacted since attention mechanisms (Xu et al., 2015; Vaswani et al., 2017; Wang et al., 2018) and the emergence of residual connections (He et al., 2016) were introduced to train deep networks. The design of the BBS $\left(B_{i}\right)$is built on the combination of these concepts. As shown in Figure 6, BBS consist of a multiple local residual learning (LRL) skip connection block and a FAB. Local residual learning permits low-frequency details to be bypassed through multiple local residual learning, allowing the main network to learn discriminatively useful information. The combination of several basic block structures with skip connections increases the depth and capability of the ARB in overcoming training challenges.

By implementing a two-layer convolutional network at the end of the ARB network (as shown in Figure 4) and employing a long-skip connection global residual learning module as a recovery strategy to restore the input camouflage image.

### 3.6 Feature fusion attention strategy

Shallow feature information can often be difficult to retain as the network gets deeper. U-Net (Ronneberger et al., 2015) and other networks strive to fuse different level features of shallow and deep information. As depicted in Figure 4, feature maps produced by the 𝐺 group architecture in the channel direction are concatenated. Following the FAB weighting strategy, the retained low-level features with edge information in the shallow layer that preserve spatial details for establishing object boundaries are fed into deep layers, allowing the ARB network (ARB-Net) to focus more on semantic information like high-frequency textures for hidden objects scene visibility in real-world scenarios.

### 3.7 Loss function

According to Lim et al. (2017), training with L1 loss often outperformed training with L2 loss for image restoration tasks. Following the same strategy, we adopted L1 loss as our default loss function for training the ARB-Net. The total loss function L is:


(9) $$L\left(\Theta \right)=\frac{1}{N}\;\sum_{i=1}^{N}\left\|I_{gt}^{i}-ARB\left(I_{c}^{i}\right)\right\|$$

where Θ represents the ARB-Net parameters, $I_{gt}^{i}$ stands for ground truth, and $I_{c}^{i}$ stands for the real-world camouflaged input image. The proposed ARB-Net extends the hyperparameters detailed in Qin et al. (2020), encompassing vital parameters like image size, learning rate, optimizer, batch size, and loss function. The selection process for the Adaptive Restoration Block (ARB) was meticulously executed through a systematic approach combining experimentation, domain knowledge, and optimization techniques. Leveraging our understanding of camouflage object detection and image restoration, we meticulously fine-tuned the hyperparameters to meet the unique demands of the task. Through iterative adjustments and rigorous validation of test data, we identified the most effective configuration for the ARB. This comprehensive approach ensures that the ARB-Net is finely tuned to excel in the intricate domain of camouflage object detection, enhancing its performance and applicability in real-world scenarios.

### 3.8 Cascaded detection block

#### 3.8.1 Sensory module (SM)

According to a neuroscience study by Langley et al. (1996), when prey indiscriminately hides in the background, selective search attention (Riley and Roitblat, 2018) plays a significant role in the predatory sensory mechanism to reduce non-prey details, thus saving computational time. To take advantage of the sensory mechanism, search attention is used in the initial feature learning to select and aggregate semantic features from the restored camouflage image $I_{ARB}$ in the previous section.

Given an input image $I_{ARB}\;\epsilon \;R^{W\;x\;H\;x3}$ (the output of the ARB) a set of features $\left\{f_{k},k\;\epsilon \;\left\{1,2,3,4,5\right\}\right\}$is extracted from the ResNet-50 (He et al., 2016) backbone architecture. The resolution of each feature $f_{k}$is $\frac{H}{2^{k}}\;x$ $\frac{W}{2^{k}}$, $k=\left\{4,4,8,16,32\right\}\text{.}$Studies by Lin et al. (2017) demonstrated that high-level features in deep layers keep semantic information for finding objects, whereas low-level features in shallow layers preserve spatial details for establishing object boundaries. Based on the property of neural networks, extracted features are categorized as low-level $\left\{X_{0},X_{1}\right\}$, intermediate-level $\left\{X_{2}\right\}$, and high-level features $\left\{X_{3},X_{4}\right\}\;\text{,}$which are later fused through concatenation, up-sampling, and down-sampling operations; thereafter, by leveraging a dense convolutional network strategy of (Huang et al., 2017) to preserve more information from different layers and then use a modified receptive field (Liu and Huang, 2018) block to enlarge the receptive field and output a set of enhanced features.

#### 3.8.2 Identification module (IM)

In the identification module, disguised objects need to be precisely identified using the output features obtained from the previous sensory module. Following the identification network of (Fan et al., 2020a), our final context-aware camouflaged object prediction maps with refined boundaries are generated.

## 4 Results

To demonstrate the generality of our newly proposed DiCANet COD model, the ARB-Net goes through a fine-tuning stage with different key network parameters and is trained on local image patches to perform restoration for more complex image background scenarios. For optimal results that preserve the camouflaged object’s latent spectral content and structural details, the Group Structure 𝐺 and each Basic Block Structure 𝐵 are set to 3 and 19 respectively, in the ARB. The filter size for all convolution layers is set to 3∗3, except for the Channel Attention, whose kernel size is 1∗1. Additionally, all feature maps maintain a fixed size except for the Channel Attention module. Each Group Structure outputs 64 filters.

## 5 Discussion

### 5.1 Experimental settings

#### 5.1.1 Training/Testing details

ARB-Net builds on the same training settings of (Qin et al., 2020). Following the same hyperparameter configurations of (Fan et al., 2020a) for CDB. We evaluate the DiCANet models on the whole CHAMELEON (Skurowski et al., 2018) and test sets of CAMO (Le et al., 2019), and COD10K (Fan et al., 2020a). The entire experiment was executed on a 2.2 GHz dual-core Intel Core i7 CPU with 8 GB of RAM using Google COLAB as our working interface. Evaluation Metrics: We adopt four benchmark evaluation metrics to evaluate the performance of the DiCANet model including S-measure (Fan et al., 2017), mean E-measure (Fan et al., 2018), weighted F-measure (Margolin et al., 2014), and Mean Absolute Error.

### 5.2 Baseline models

To demonstrate the robustness of DiCANet, this research selected 13 strong baseline methods that adopted ResNet50 (He et al., 2016) as the backbone network for feature extraction and achieved SOTA performance in related fields, namely GOD and SOD: object detection FPN (Lin et al., 2017); semantic segmentation PSPNet (Zhao et al., 2017); instance segmentation Mask RCNN (He et al., 2017), HTC (Chen et al., 2019), and MSRCNN (Huang et al., 2019); medical image segmentation UNet++ (Zhou et al., 2018) and PraNet (Fan et al., 2020b); salient object detection PiCANet (Liu et al., 2018) BASNet (Qin et al., 2019), CPD (Wu et al., 2019), PFANet (Zhao and Wu, 2019), EGNet (Zhao J. X. et al., 2019), and camouflaged object segmentation SINet (Fan et al., 2020a).

### 5.3 Quantitative comparison

Table 1 summarizes the quantitative results of different baselines on three standard COD datasets. The proposed model achieved the highest values for the evaluation metrics, which indicates superior performance.

**Table 1:** Quantitative comparison in terms of $S_{\propto }$, $E_{\phi },F_{\beta }^{\omega },\mathrm{and}\;M$ on three benchmark COD datasets (Fan et al., 2020a).

Baseline models	CHAMELEON				CAMO – Test				COD10K – Test			
	$S_{\propto }↑$	$E_{\phi }↑$	$F_{\beta }^{\omega }↑$	M↓	$S_{\propto }↑$	$E_{\phi }↑$	$F_{\beta }^{\omega }↑$	M↓	$S_{\propto }↑$	$E_{\phi }↑$	$F_{\beta }^{\omega }↑$	M↓
FPN	0.794	0.783	0.590	0.075	0.684	0.677	0.483	0.131	0.697	0.691	0.411	0.075
MaskRCNN	0.643	0.778	0.518	0.099	0.574	0.715	0.430	0.151	0.613	0.748	0.402	0.080
PSPNet	0.773	0.758	0.555	0.085	0.663	0.659	0.455	0.139	0.678	0.680	0.377	0.080
UNet++	0.695	0.762	0.501	0.094	0.599	0.653	0.392	0.149	0.623	0.672	0.350	0.086
PiCANet	0.769	0.749	0.536	0.085	0.609	0.584	0.356	0.156	0.649	0.643	0.322	0.090
MSRCNN	0.637	0.686	0.443	0.091	0.617	0.669	0.454	0.133	0.641	0.706	0.419	0.073
BASNet	0.687	0.721	0.474	0.118	0.618	0.661	0.413	0.159	0.634	0.678	0.365	0.105
PFANet	0.679	0.648	0.378	0.144	0.659	0.622	0.391	0.172	0.636	0.618	0.286	0.128
CPD	0.853	0.866	0.706	0.052	0.726	0.729	0.550	0.115	0.747	0.770	0.508	0.059
HTC	0.517	0.489	0.204	0.129	0.476	0.442	0.174	0.172	0.548	0.520	0.221	0.088
EGNet	0.848	0.870	0.702	0.050	0.732	0.768	0.583	0.104	0.737	0.779	0.509	0.056
PraNet	0.860	0.907	0.763	0.044	0.769	0.824	0.663	0.094	0.789	0.861	0.629	0.045
SINet	0.869	0.891	0.740	0.044	0.751	0.771	0.606	0.100	0.771	0.806	0.551	0.051
DiCANet (Ours)	**0.871**	**0.950**	**0.805**	**0.034**	0.747	**0.828**	**0.647**	**0.091**	**0.775**	**0.872**	**0.629**	**0.043**

The best scores are highlighted in bold. t indicates the higher the score the better, and ↓: the lower the better.

For the CAMO dataset, comparing DiCANet model with the top two performing baselines: PraNet and SINet, the proposed method improved by 0.003 and 0.009, respectively in terms of M, and by 0.057 and 0.041, respectively, in terms of $E_{\phi }$and $F_{\beta }^{\omega }$. Although DiCANet achieved a low structural similarity score $S_{\propto }$, accurate predictions with high integrity of preserved edge details and clear boundaries were still achieved. Similarly, when compared with the edge boundary models, e.g., EGNet and PFANet, our DiCANet improves $E_{\phi }$ and $F_{\beta }^{\omega }$ by (0.08 and 0.103) and (0.302 and 0.427), respectively, while drastically reducing MAE error by 0.016 and 0.110 for the CHAMELEON dataset. DiCANet achieved a significant improvement in $S_{\propto }$ of 0.011 compared with the best model PraNet. Interestingly, for the most challenging dataset, COD10K, DiCANet outperformed the competition in prediction accuracy for all metrics and boosted performance to a new SOTA.

### 5.4 Qualitative comparison

**Figure 7:**
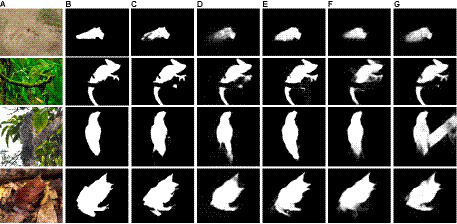
Camouflaged objects segmentation results. **(A)** Image, **(B)** GT, **(C)** DiCANet, **(D)** SINet, **(E)** PraNet, **(F)** EGNet, **(G)** CPD.

Figure 7 shows the qualitative comparison of the camouflaged prediction map of DiCANet against the top four cutting-edge models. Row 1 to row 2, (top to bottom) are examples from CHAMELEON datasets; row 3 are examples from CAMO datasets; row 4 is an example from COD10K’s super-class: amphibious. It is evident that DiCANet outperforms all competing models and provides the best prediction that is the closest to ground truth (best viewed when zoomed).

Noncamouflaged regions are consistently included in the results of the compared methods, while some details of camouflaged objects are neglected. In contrast, the competing models inaccurately detect disguised objects and provide unreliable visual results. The proposed model demonstrated excellent performance in locating concealed objects accurately, with rich, fine details in predictions and clear boundaries. Additionally, our method captures the object boundaries quite well due to the power of ARB’s adaptive weighing mechanism and feature fusion strategy.

#### 5.4.1 Failure case

**Figure 8:**
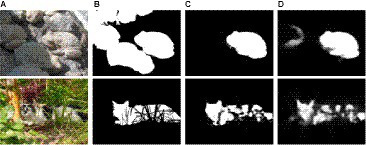
Failure cases of our DiCANet. **(A)** Images, **(B)** GT, **(C)** Ours, **(D)** SINet.

Despite achieving satisfactory quantitative performance and setting a record in the COD task, the proposed DiCANet framework exhibits limitations in specific scenarios as shown in Figure 8. When dealing with multiple camouflaged objects grouped closely together (*row 1*), DiCANet might struggle to accurately predict the number of objects. This limitation can be attributed to the network’s limited prior knowledge in handling scenes with a specific number of objects. The complicated topological structures (*row 2*) with dense details can also pose challenges for DiCANet due to background complexity distraction. This complexity overwhelms the attention mechanisms, diverting focus from the camouflaged objects. Additionally, the intricate details in the background could share similar features with the camouflage patterns, making it difficult to distinguish the camouflaged object from its surroundings. These limitations provide valuable insights and potential areas for future investigation. By tackling these challenges and exploring novel approaches, researchers can create more resilient COD systems capable of managing even the most intricate and challenging scenarios.

#### 5.4.2 Ablation study

**Figure 9:**
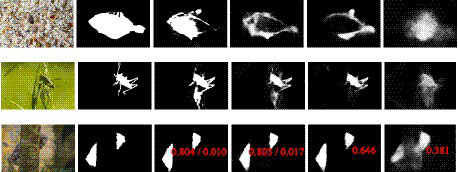
Visual comparison with top three baselines on COD10K $\left(S_{\propto }/M\right)$.

To further demonstrate the superiority of DiCANet architecture with previous state-of-the-art methods, we conducted an ablation study by considering challenging camouflage scenarios (Figure 9). The study observes that DiCNet consistently shows distinctive detection and segmentation of concealed objects in challenging natural scenarios, such as partial occlusion (1st row), weak object/background contrast (2nd row), and strong background descriptor (3rd row). Meanwhile, the structural similarity 𝑺∝ scores (*in red*) of DiCANet are much higher and with a minimal error (*in red*) compared to the competitors, which further demonstrates the superiority of our method. We can also clearly see that the combination of the proposed adaptive ARB-Net and *Feature Fusion Attention Strategy* has significantly elevated our results to an exceptional level.

## 6 Conclusion

This paper presents Discriminative Context-Aware Network (DiCANet), a novel joint learning framework for detecting concealed objects with refined edges. The proposed model leverages two key components: the ARB-Net and the CDB. To improve the camouflage scene visibility, we employed ARB-Net to adaptively generate different attention weights for each channel-and pixel-wise feature and strategically fuse the feature maps to expand the discriminative power and representative ability of the convolution networks. To drive camouflage object localization and segmentation performance, we employed the CDB module. Based on the ARB and CDB modules, a context-aware network that effectively aims to pay more attention to local contextual information to evaluate the objectivity of the camouflage prediction map was proposed. Extensive experiments show that mining distinctive information can overcome the difficulties of both SOD and COD tasks with superior performance; thus, DiCANet outperforms SOTA methods under the commonly used evaluation metrics and deserves further exploration in other related computer vision tasks.

## Data availability statement

The original contributions presented in the study are included in the article/supplementary material, further inquiries can be directed to the corresponding authors.

## Author contributions

CI: Conceptualization, Data curation, Formal analysis, Investigation, Methodology, Resources, Software, Validation, Visualization, Writing – original draft, Writing – review & editing. NM: Data curation, Formal analysis, Investigation, Methodology, Project administration, Resources, Software, Validation, Visualization, Writing – original draft, Writing – review & editing. NB: Conceptualization, Data curation, Formal analysis, Investigation, Project administration, Resources, Validation, Visualization, Writing – original draft, Writing – review & editing. SA: Data curation, Formal analysis, Funding acquisition, Investigation, Project administration, Resources, Validation, Visualization, Writing – original draft, Writing – review & editing. FE: Conceptualization, Data curation, Formal analysis, Investigation, Methodology, Project administration, Resources, Supervision, Validation, Visualization, Writing – original draft, Writing – review & editing.

## References

[ref1] Abdelhamed A. Lin S. Brown M. S. (2018). A high-quality denoising dataset for smartphone cameras, in “Proceedings of the IEEE conference on computer vision and pattern recognition”, pp. 1692–1700.

[ref3] Chen L. Chu X. Zhang X. Sun J. (2022). Simple baselines for image restoration. arXiv. doi: 10.48550/arXiv.2204.04676

[ref4] Chen K. Pang J. Wang J. Xiong Y. Li X. Sun S.. (2019). Hybrid task cascade for instance segmentation. Proceedings of the IEEE/CVF Conference on Computer Vision and Pattern Recognition, pp. 4974–4983.

[ref5] Chu X. Chen L. Chen C. Lu X. (2021). Revisiting global statistics aggregation for improving image restoration. arXiv

[ref6] Chu H. K. Hsu W. H. Mitra N. J. Cohen-Or D. Wong T. T. Lee T. Y. (2010). Camouflage images. ACM Trans. Graph. 29:1. doi: 10.1145/1833351.1778788

[ref7] Cott H. B. (1940). Adaptive coloration in animals. Methuen, London.

[ref8] Dai T. Cai J. Zhang Y. Xia S. T. Zhang L. (2019). Second-order attention network for single image super-resolution. Proceedings of the IEEE/CVF conference on computer vision and pattern recognition, pp. 11065–11074.

[ref9] Deng J. Dong W. Socher R. Li L. J. Li K. Fei-Fei L. (2009). Imagenet: a large-scale hierarchical image database. In 2009 IEEE conference on computer vision and pattern recognition, IEEE, pp. 248–255.

[ref10] Dong B. Zhuge M. Wang Y. Bi H. Chen G. (2021). Accurate camouflaged object detection via mixture convolution and interactive fusion. arXiv. doi: 10.48550/arXiv.2101.05687

[ref11] Dosovitskiy A. Beyer L. Kolesnikov A. Weissenborn D. Zhai X. Unterthiner T.. (2020). An image is worth 16x16 words:Transformers for image recognition at scale. arXiv. doi: 10.48550/arXiv.2010.11929

[ref12] Everingham M. Eslami S. M. Van Gool L. Williams C. K. Winn J. Zisserman A. (2015). The pascal visual object classes challenge: a retrospective. Int. J. Comput. Vis. 111, 98–136. doi: 10.1007/s11263-014-0733-5

[ref13] Fan D. P. Cheng M. M. Liu Y. Li T. Borji A. Structure-measure: a new way to evaluate foreground maps. Proceedings of the IEEE international conference on computer vision. (2017), pp. 4548–4557.

[ref14] Fan D. P. Gong C. Cao Y. Ren B. Cheng M. M. Borji A. (2018). Enhanced-alignment measure for binary foreground map evaluation. arXiv. doi: 10.48550/arXiv.1805.10421

[ref15] Fan D. P. Ji G. P. Sun G. Cheng M. M. Shen J. Shao L. (2020a). Camouflaged object detection. In Proceedings of the IEEE/CVF conference on computer vision and pattern recognition, pp. 2777–2787.

[ref16] Fan D. P. Ji G. P. Zhou T. Chen G. Fu H. Shen J.. (2020b). “Pranet: Parallel reverse attention network for polyp segmentation” in International conference on medical image computing and computer-assisted intervention (Cham: Springer), 263–273.

[ref17] Fan D. P. Zhou T. Ji G. P. Zhou Y. Chen G. Fu H.. (2020c). INF-net: automatic COVID-19 lung infection segmentation from ct images. IEEE Trans. Med. Imaging 39, 2626–2637. doi: 10.1109/TMI.2020.2996645, PMID: 32730213

[ref18] Flannelly K. J. (2017). Religious beliefs, evolutionary psychiatry, and mental health in America. New York, NY: Springer.

[ref19] Forrest N. (2016). SqueezeNet: AlexNet-level accuracy with 50x fewer parameters. arXiv. doi: 10.48550/arXiv.1602.07360

[ref20] Galloway J. A. Green S. D. Stevens M. Kelley L. A. (1802). Finding a signal hidden among noise: how can predators overcome camouflage strategies? Philos. Trans. R. Soc. B 2020:20190478. 10.1098/rstb.2019.0478 PMC7331011 32420842

[ref21] Galun M. Sharon E. Basri R. Brandt A. (2003). Texture segmentation by multiscale aggregation of filter responses and shape elements. ICCV 3:716. doi: 10.1109/ICCV.2003.1238418

[ref22] Ge S. Jin X. Ye Q. Luo Z. Li Q. (2018). Image editing by object-aware optimal boundary searching and mixed-domain composition. Comput. Vis. Media 4, 71–82. doi: 10.1007/s41095-017-0102-8

[ref23] Girshick R. (2015). “Fast r-cnn” in Proceedings of the IEEE international conference on computer vision, 1440–1448.

[ref24] Girshick R. Donahue J. Darrell T. Malik J. Rich feature hierarchies for accurate object detection and semantic segmentation. In Proceedings of the IEEE conference on computer vision and pattern recognition. (2014), pp. 580–587.

[ref25] Gleeson P. Lung D. Grosu R. Hasani R. Larson S. D. (2018). c302: a multiscale framework for modelling the nervous system of *Caenorhabditis elegans*. Philos. Trans. Royal Soc. B 373:20170379. doi: 10.1098/rstb.2017.0379, PMID: 30201842 PMC6158223

[ref26] Han Q. Fan Z. Dai Q. Sun L. Cheng M. M. Liu J.. (2021). Demystifying local vision transformer: Sparse connectivity, weight sharing, and dynamic weight. arXiv 2.

[ref27] He K. Gkioxari G. Dollár P. Girshick R. (2017). Mask R-CNN. Proceedings of the IEEE international conference on computer vision, pp. 2961–2969.

[ref28] He K. Sun J. Tang X. (2010). Single image haze removal using dark channel prior. IEEE Trans. Pattern Anal. Mach. Intell. 33, 2341–2353. doi: 10.1109/TPAMI.2010.168, PMID: 20820075

[ref29] He K. Zhang X. Ren S. Sun J. (2016). Deep residual learning for image recognition. Proceedings of the IEEE conference on computer vision and pattern recognition, pp. 770–778.

[ref30] Hou J. Y. Y. H. W. (2011). Detection of the mobile object with camouflage color under dynamic background based on optical flow. Procedia Eng. 15, 2201–2205. doi: 10.1016/j.proeng.2011.08.412

[ref31] Hu J. Shen L. Sun G. (2018). Squeeze-and-excitation networks. Proceedings of the IEEE conference on computer vision and pattern recognition, 7132–7141.

[ref32] Huang Z. Huang C. Wang X. (2019). Mask scoring R-CNN. CVPR, 6409–6418. doi: 10.48550/arXiv.1903.00241

[ref33] Huang G. Liu Z. Van Der Maaten L. Weinberger K. Q. (2017). Densely connected convolutional networks. Proceedings of the IEEE conference on computer vision and pattern recognition, pp. 4700–4708.

[ref34] Hussain N. Khan M. A. Kadry S. Tariq U. Mostafa R. R. Choi J. I.. (2021). Intelligent deep learning and improved whale optimization algorithm based framework for object recognition. Hum. Cent. Comput. Inf. Sci 11:2021. doi: 10.22967/HCIS.2021.11.034

[ref35] Ismail Fawaz H. Forestier G. Weber J. Idoumghar L. Muller P. A. (2019). Deep learning for time series classification: a review. Data Min. Knowl. Disc. 33, 917–963. doi: 10.1007/s10618-019-00619-1

[ref36] Ji G. P. Zhu L. Zhuge M. Fu K. (2022). Fast camouflaged object detection via edge-based reversible re-calibration network. Pattern Recogn. 123:108414. doi: 10.1016/j.patcog.2021.108414

[ref38] Kirillov A. He K. Girshick R. Rother C. Dollár P. (2019). Panoptic segmentation. Proceedings of the IEEE/CVF conference on computer vision and pattern recognition, pp. 9404–9413.

[ref39] Lamdouar H. Yang C. Xie W. Zisserman A. (2020). Betrayed by motion: Camouflaged object discovery via motion segmentation. Proceedings of the Asian Conference on Computer Vision.

[ref40] Langley C. M. Riley D. A. Bond A. B. Goel N. (1996). Visual search for natural grains in pigeons (*Columba livia*): search images and selective attention. J. Exp. Psychol. Anim. Behav. Process. 22, 139–151. PMID: 8618099 10.1037//0097-7403.22.2.139

[ref41] Le T. N. Cao Y. Nguyen T. C. Le M. Q. Nguyen K. D. Do T. T.. (2021). Camouflaged instance segmentation in-the-wild: dataset, method, and benchmark suite. IEEE Trans. Image Process. 31, 287–300. doi: 10.1109/TIP.2021.3130490 34855592

[ref42] Le T. N. Nguyen T. V. Nie Z. Tran M. T. Sugimoto A. (2019). Anabranch network for camouflaged object segmentation. Comput. Vis. Image Underst. 184, 45–56. doi: 10.1016/j.cviu.2019.04.006

[ref43] Le T. N. Ono S. Sugimoto A. Kawasaki H. (2020). Attention R-CNN for accident detection. In 2020 IEEE intelligent vehicles symposium (IV), pp. 313–320.

[ref44] Lim B. Son S. Kim H. Nah S. Lee M., (2017). Enhanced deep residual networks for single image super-resolution. Proceedings of the IEEE conference on computer vision and pattern recognition workshops, pp. 136–144.

[ref45] Lin M. Chen Q. Yan S. (2014). Network in Network. arXiv. doi: 10.48550/arXiv.1312.4400

[ref46] Lin T. Y. Dollár P. Girshick R. He K. Hariharan B. Belongie S. (2017). Feature pyramid networks for object detection. Proceedings of the IEEE conference on computer vision and pattern recognition, pp. 2117–2125.

[ref47] Lin T. Y. Maire M. Belongie S. Hays J. Perona P. Ramanan D.. (2014). “Microsoft coco: Common objects in context” in European conference on computer vision (Cham: Springer), 740–755.

[ref48] Liu N. Han J. Yang M. H. (2018). Picanet: learning pixel-wise contextual attention for saliency detection. Proceedings of the IEEE conference on computer vision and pattern recognition, pp. 3089–3098.

[ref49] Liu S. Huang D. (2018). Receptive field block net for accurate and fast object detection. In Proceedings of the European conference on computer vision, pp. 385–400.

[ref50] Liu Z. Huang K. Tan T. (2012). Foreground object detection using top-down information based on EM framework. IEEE Trans. Image Process. 21, 4204–4217. doi: 10.1109/TIP.2012.2200492, PMID: 22645266

[ref51] Liu Z. Mao H. Wu C. Y. Feichtenhofer C. Darrell T. Xie S. (2022). A convnet for the 2020s. In Proceedings of the IEEE/CVF conference on computer vision and pattern recognition, pp. 11976–11986.

[ref52] Liu C. Yuen J. Torralba A. (2010). Sift flow: dense correspondence across scenes and its applications. IEEE Trans. Pattern Anal. Mach. Intell. 33, 978–994. doi: 10.1109/TPAMI.2010.147 20714019

[ref54] Machine Learning Mastery. (2019) A Gentle Introduction to Pooling Layers for Convolutional Neural Networks. Available at: https://machinelearningmastery.com/crash-course-convolutional-neural-networks/

[ref55] Margolin R. Zelnik-Manor L. Tal A. (2014). How to evaluate foreground maps?. Proceedings of the IEEE conference on computer vision and pattern recognition, pp. 248–255.

[ref56] Mei H. Ji G. P. Wei Z. Yang X. Wei X. Fan D. P. (2021). Camouflaged object segmentation with distraction mining. In Proceedings of the IEEE/CVF conference on computer vision and pattern recognition, pp. 8772–8781.

[ref57] Merilaita S. Scott-Samuel N. E. Cuthill I. C. (2017). How camouflage works. Philos. Trans. Royal Soc. B 372:20160341. doi: 10.1098/rstb.2016.0341, PMID: 28533458 PMC5444062

[ref58] Nah S. Hyun Kim T. Lee M.. (2017). Deep multi-scale convolutional neural network for dynamic scene deblurring. In Proceedings of the IEEE conference on computer vision and pattern recognition, pp. 3883–3891.

[ref59] Naqvi S. M. A. Shabaz M. Khan M. A. Hassan S. I. (2023). Adversarial attacks on visual objects using the fast gradient sign method. J Grid Comput 21:52. doi: 10.1007/s10723-023-09684-9

[ref60] Niu B. Wen W. Ren W. Zhang X. Yang L. Wang S.. (2020). “Single image super-resolution via a holistic attention network” in European conference on computer vision (Cham: Springer), 191–207.

[ref61] Pan Y. Chen Y. Fu Q. Zhang P. Xu X. (2011). Study on the camouflaged target detection method based on 3D convexity. Mod. Appl. Sci. 5:152. doi: 10.5539/mas.v5n4p152

[ref62] Qadeer N. Shah J. H. Sharif M. Khan M. A. Muhammad G. Zhang Y. D. (2022). Intelligent tracking of mechanically thrown objects by industrial catching robot for automated in-plant logistics 4.0. Sensors 22:2113. doi: 10.3390/s22062113, PMID: 35336292 PMC8955428

[ref63] Qin X. Wang Z. Bai Y. Xie X. Jia H. (2020). FFA-net: feature fusion attention network for single image dehazing. Proc. AAAI Conf. Artif. Intel. 34, 11908–11915. doi: 10.1609/aaai.v34i07.6865

[ref64] Qin X. Zhang Z. Huang C. Gao C. Dehghan M. Jagersand M. (2019). Basnet: boundary-aware salient object detection. Proceedings of the IEEE/CVF conference on computer vision and pattern recognition, pp. 7479–7489.

[ref65] Ren S. He K. Girshick R. Sun J. (2015). Faster R-CNN: towards real-time object detection with region proposal networks. Adv. Neural Inf. Proces. Syst. 28, 1137–1149. doi: 10.48550/arXiv.1506.01497 27295650

[ref66] Ren S. He K. Girshick R. Sun J. (2017). Faster R-CNN: towards real-time object detection with region proposal networks. IEEE Trans. Pattern Anal. Mach. Intell. 39, 1137–1149. doi: 10.1109/TPAMI.2016.2577031, PMID: 27295650

[ref67] Rida I. Al-Maadeed N. Al-Maadeed S. Bakshi S. (2020). A comprehensive overview of feature representation for biometric recognition. Multimed. Tools Appl. 79, 4867–4890. doi: 10.1007/s11042-018-6808-5

[ref68] Riley D. A. Roitblat H. L. (2018). Selective attention and related cognitive processes in pigeons. Cogn. Proces. Anim. Behav., 249–276. doi: 10.4324/9780203710029-9

[ref69] Ronneberger O. Fischer P. Brox T. (2015). “U-net: convolutional networks for biomedical image segmentation” in International conference on medical image computing and computer-assisted intervention (Cham: Springer), 234–241.

[ref70] Sengottuvelan P. Wahi A. Shanmugam A. (2008). Performance of decamouflaging through exploratory image analysis. 2008 first international conference on emerging trends in engineering and technology, pp. 6–10.

[ref71] Shotton J. Winn J. Rother C. Criminisi A. (2006). “Textonboost: joint appearance, shape and context modeling for multi-class object recognition and segmentation” in European conference on computer vision (Berlin: Springer), 1–15.

[ref72] Skurowski P. Abdulameer H. Błaszczyk J. Depta T. Kornacki A. Kozieł P. (2018), Animal camouflage analysis: CHAMELEON database. *Unpublished manuscript*, 2, p. 7.

[ref73] Song L. Geng W. (2010). A new camouflage texture evaluation method based on WSSIM and nature image features. 2010 international conference on multimedia technology, pp. 1–4.

[ref74] Stevens M. Merilaita S. (2009). Animal camouflage: current issues and new perspectives. Philos. Trans. Royal Soc. B 364, 423–427. doi: 10.1098/rstb.2008.0217, PMID: 18990674 PMC2674078

[ref75] Stevens M. Ruxton G. D. (2019). The key role of behaviour in animal camouflage. Biol. Rev. 94, 116–134. doi: 10.1111/brv.12438, PMID: 29927061 PMC6378595

[ref76] Talas L. Baddeley R. J. Cuthill I. C. (2017). Cultural evolution of military camouflage. Philos. Trans. Royal Soc. B 372:20160351. doi: 10.1177/10482911211032971, PMID: 28533466 PMC5444070

[ref77] Thayer G. H. (1918). Concealing-coloration in the animal kingdom: An exposition of the laws of disguise through color and pattern. New York: Macmillan Company.

[ref78] Ulyanov D. Vedaldi A. Lempitsky V. (2018). Deep image prior. Proceedings of the IEEE conference on computer vision and pattern recognition (pp. 9446–9454).

[ref79] Vaswani A. Shazeer N. Parmar N. Uszkoreit J. Jones L. Gomez A. N.. (2017). Attention is all you need. Adv. Neural Inf. Proces. Syst. 30, 5998–6008. doi: 10.48550/arXiv.1706.03762

[ref80] Wang T. Borji A. Zhang L. Zhang P. Lu H. (2017). A stagewise refinement model for detecting salient objects in images. In Proceedings of the IEEE international conference on computer vision, pp. 4019–4028.

[ref81] Wang Z. Cun X. Bao J. Zhou W. Liu J. Li H. (2022). Uformer: a general u-shaped transformer for image restoration. In Proceedings of the IEEE/CVF conference on computer vision and pattern recognition, pp. 17683–17693.

[ref82] Wang X. Girshick R. Gupta A. He K. (2018). Non-local neural networks. Proceedings of the IEEE conference on computer vision and pattern recognition, pp. 7794–7803.

[ref83] Waqas Zamir S. Arora A. Khan S. Hayat M. Shahbaz Khan F. Yang M. H. (2021). Restormer: efficient transformer for high-resolution image restoration. arXiv:2111. doi: 10.48550/arXiv.2111.09881

[ref84] Woo S. Park J. Lee J. Y. Kweon I. S. (2018). CBAM: convolutional block attention module. Proceedings of the European conference on computer vision (ECCV), pp. 3–19.

[ref85] Wu Y. H. Gao S. H. Mei J. Xu J. Fan D. P. Zhang R. G.. (2021). Jcs: an explainable COVID-19 diagnosis system by joint classification and segmentation. IEEE Trans. Image Process. 30, 3113–3126. doi: 10.1109/TIP.2021.3058783, PMID: 33600316

[ref86] Wu Z. Su L. Huang Q. (2019). Cascaded partial decoder for fast and accurate salient object detection. In Proceedings of the IEEE/CVF conference on computer vision and pattern recognition, pp. 3907–3916.

[ref87] Xu K. Ba J. Kiros R. Cho K. Courville A. Salakhudinov R.. (2015). Show, attend and tell: Neural image caption generation with visual attention. International conference on machine learning, pp. 2048–2057

[ref88] Xue F. Yong C. Xu S. Dong H. Luo Y. Jia W. (2016). Camouflage performance analysis and evaluation framework based on features fusion. Multimed. Tools Appl. 75, 4065–4082. doi: 10.1007/s11042-015-2946-1

[ref89] Zamir S. W. Arora A. Khan S. Hayat M. Khan F. S. Yang M. H. (2022). Restormer: efficient transformer for high-resolution image restoration. In Proceedings of the IEEE/CVF conference on computer vision and pattern recognition, pp. 5728–5739.

[ref90] Zamir S. W. Arora A. Khan S. Hayat M. Khan F. S. Yang M. H.. (2020). “Learning enriched features for real image restoration and enhancement” in European conference on computer vision (Cham: Springer), 492–511.

[ref91] Zhang Y. Li K. Li K. Wang L. Zhong B. Fu Y. (2018). Image super-resolution using very deep residual channel attention networks. In Proceedings of the European conference on computer vision (ECCV), pp. 286–301.

[ref92] Zhao J. X. Liu J. J. Fan D. P. Cao Y. Yang J. Cheng M. M. (2019). EGNet: edge guidance network for salient object detection. Proceedings of the IEEE/CVF international conference on computer vision, pp. 8779–8788.

[ref93] Zhao H. Shi J. Qi X. Wang X. Jia J. (2017). Pyramid scene parsing network. Proceedings of the IEEE conference on computer vision and pattern recognition, pp. 2881–2890.

[ref94] Zhao T. Wu X. (2019). Pyramid feature attention network for saliency detection. In Proceedings of the IEEE/CVF conference on computer vision and pattern recognition, pp. 3085–3094.

[ref95] Zhao Z. Q. Zheng P. Xu S. T. Wu X. (2019). Object detection with deep learning: a review. IEEE Trans. Neural Netw. Learn. Syst. 30, 3212–3232. doi: 10.1109/TNNLS.2018.2876865 30703038

[ref96] Zheng Y. Zhang X. Wang F. Cao T. Sun M. Wang X. (2018). Detection of people with camouflage pattern via dense deconvolution network. IEEE Signal Proces. Lett. 26, 29–33. doi: 10.1109/LSP.2018.2825959

[ref97] Zhou Z. Rahman Siddiquee M. M. Tajbakhsh N. Liang J. (2018). “UNET++: A nested U-Net architecture for medical image segmentation” in Deep learning in medical image analysis and multimodal learning for clinical decision support (Cham: Springer), 3–11. 10.1007/978-3-030-00889-5_1 PMC7329239 32613207

[ref98] Zhu J. Zhang X. Zhang S. Liu J. (2021). Inferring camouflaged objects by texture-aware interactive guidance network. Proceedings of the AAAI Conference on Artificial Intelligence, 35, pp. 3599–3607.

